# Ketone Bodies in Neurological Diseases: Focus on Neuroprotection and Underlying Mechanisms

**DOI:** 10.3389/fneur.2019.00585

**Published:** 2019-06-12

**Authors:** Huajun Yang, Wei Shan, Fei Zhu, Jianping Wu, Qun Wang

**Affiliations:** ^1^Department of Neurology, Beijing Tiantan Hospital, Capital Medical University, Beijing, China; ^2^National Center for Clinical Medicine of Neurological Diseases, Beijing, China; ^3^Beijing Institute for Brain Disorders, Beijing, China; ^4^Advanced Innovation Center for Human Brain Protection, Capital Medical University, Beijing, China

**Keywords:** ketone bodies, ketogenic diet, neuroprotection, neurological diseases, underlying mechanisms

## Abstract

There is growing evidence that ketone bodies, which are derived from fatty acid oxidation and usually produced in fasting state or on high-fat diets have broad neuroprotective effects. Although the mechanisms underlying the neuroprotective effects of ketone bodies have not yet been fully elucidated, studies in recent years provided abundant shreds of evidence that ketone bodies exert neuroprotective effects through possible mechanisms of anti-oxidative stress, maintaining energy supply, modulating the activity of deacetylation and inflammatory responses. Based on the neuroprotective effects, the ketogenic diet has been used in the treatment of several neurological diseases such as refractory epilepsy, Parkinson's disease, Alzheimer's disease, and traumatic brain injury. The ketogenic diet has great potential clinically, which should be further explored in future studies. It is necessary to specify the roles of components in ketone bodies and their therapeutic targets and related pathways to optimize the strategy and efficacy of ketogenic diet therapy in the future.

## Introduction

Ketone bodies (KBs) are considered as an alternative source of energy supply (1–3). Ketone body (KB) metabolism in humans has been a significant source of fuel of the brain in nutrient deprivation state. In humans, KBs are involved in a variety of important metabolic pathways such as fatty acid β-oxidation (FAO), gluconeogenesis, the tricarboxylic acid (TCA) cycle, *de novo* lipogenesis, and sterol biosynthesis (2, 4). Also, these are produced mainly in the liver from FAO-derived acetyl-CoA and transported to the extrahepatic tissues for terminal oxidation. This metabolic mechanism provides an alternative source of energy, especially under fasting state, during which the availability of carbohydrate decreases while the availability of fatty acid increases (4, 5). More specifically, KBs are prone to exert as a significant source of fuel for extrahepatic tissues under a group of physiological conditions, including fasting, starvation, post-exercise, low carbohydrate diets, pregnancy, and neonatal period (6).

The ketogenic diet (KD) is defined as a high-fat, low-carbohydrate diet with appropriate amounts of protein, vitamins, and minerals. This diet encourages the body to consume fats easily rather than carbohydrates under normal physiological conditions, carbohydrates in food break down into glucose and are transported around the body to supply energy. Glucose is considered an especially important source of fuel in the brain. However, if small amounts of carbohydrate are present in the diet, the fat will be converted into fatty acids and then KBs in the liver. These are then passed into the brain, replacing glucose as an energy source. The elevated levels of KBs in the blood, a state is known as ketosis, induces a therapeutic effect in several medical conditions (7). KD is primarily used in the treatment of difficult-to-control (refractory) epilepsy in children (8, 9). Besides its use in epilepsy, it has been studied in various neurological disorders such as Alzheimer's disease (AD), Parkinson's disease (PD), stroke, neurotrauma, brain tumors, amyotrophic lateral sclerosis, autism, headache, pain, and sleep disorders (7).

Although the clinical efficacy of KD therapy is widely recognized, there are still speculations about its potential mechanisms for many years, which are not fully clarified yet. Early clinical observations revealed that the mechanism of KD therapy is associated with dehydration and acidosis (10, 11). However, few pieces of evidence have shown that dehydration or fluid restriction is associated with the therapeutic effect of KD. In terms of acidosis, scholars believed that KD-induced pH changes might directly affect the ion channels and neurotransmitter receptors, exerting therapeutic effects (12). Recent studies now highlighted the important roles for KBs in the treatment of several neurological diseases (13–16). A series of potential therapeutic mechanisms of KBs have been proposed. Among these mechanisms, the neuroprotective effects of KBs have attracted the attention of researchers in recent years. Hence, in this review, we discussed the underlying mechanisms of the neuroprotective effects of KBs and the application of KD in different neurological diseases based on neuroprotection.

## Overview of Ketone Body (KB) Metabolism

In physiological states such as starvation, the liver metabolizes fatty acids to produce ketones for energy supply. Ketogenesis primarily occurs in the hepatic mitochondrial matrix at rates which are proportional to total fat oxidation. Fatty acids undergo β-oxidation in the liver to produce large amounts of acetyl-CoA that enters the tricarboxylic acid cycle, and the remaining is converted into KBs (6). After the transportation of acyl chains across the mitochondrial membranes and underwent β-oxidation, the mitochondrial isoform of 3-hydroxymethyl glutaryl-CoA synthase catalyzes acetoacetyl-CoA and acetyl-CoA to generate HMG-CoA. Then HMG-CoA lyase cleaves HMG-CoA to acetyl-CoA and acetoacetate (ACA). ACA, in turn, is reduced to D-β-hydroxybutyrate (D-βHB) by phosphatidylcholine-dependent mitochondrial D-βHB dehydrogenase (BDH1) in an NAD+/NADH-coupled reaction (17, 18). The ratio of ACA/D-βHB is directly proportional to the ratio of mitochondrial NAD+/NADH (19). ACA is also able to decarboxylate to acetone spontaneously, which accounts for the source of the sweet odor in patients suffering from ketoacidosis (20). Normally, acetone that is produced in small amounts can be exhaled through the lungs, while ACA and D-βHB enter the blood circulation to provide energy for extrahepatic tissues. In classical KD, the ratio of fat to carbohydrate and protein is 4:1, which significantly reduces the intake of carbohydrates (21). Thus, KBs have become the primary source of energy to cell metabolism instead of glucose.

## Neuroprotective Effects of KB and Possible Underlying Mechanisms

**Figure 1:**
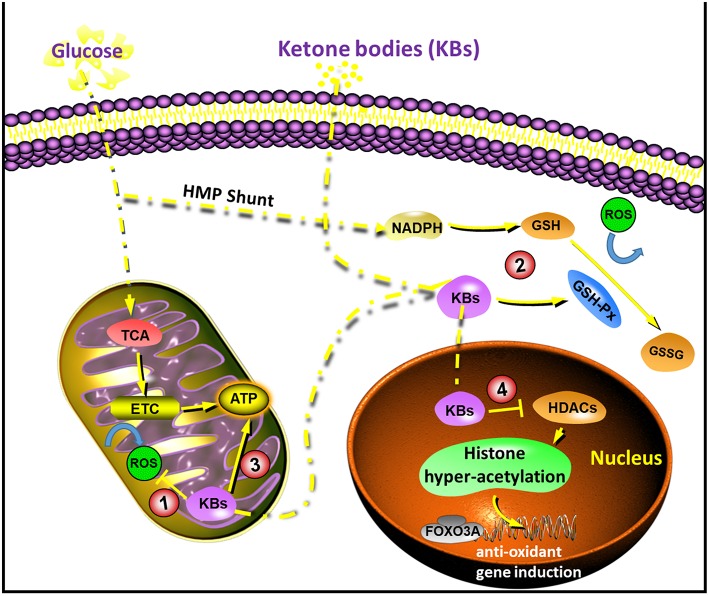
Diagram of the sites of action that underlie in the neuroprotection by ketone bodies (KBs). (1) KBs reduces NAD couple, which decreases ROS production; (2) KBs activate GSH-Px, which enhances ROS elimination; (3) KBs increase ATP concentration; (4) KBs inhibit HDACs, which increases endogenous anti-oxidants. ETC, electron transport chain; FOXO3A, forkhead box O3A; GSH-Px, glutathione peroxidase; HDACs, histone deacetylases; ROS, reactive oxygen species; TCA, tricarboxylic acid.

With the increasing research on KBs and KD, the application of KD in patients with neurological diseases has gradually become one of the research focuses in recent years. Although KD is used for treating a group of neurological diseases, the underlying mechanism remained uncertain. Recently, the neuroprotective effects of KBs have attracted more and more attention. This is because most of the neurons do not effectively generate high-energy phosphates from fatty acids, but KBs undergo oxidation in short supply of carbohydrates (22, 23). The neuroprotective effects of KBs are considered especially important (2, 24, 25). Data from recent years suggested that KBs exert their neuroprotective effects through the following possible mechanisms (Figure 1).

### Anti-oxidative Stress

Oxidative stress is generally considered as a state in which the reactive oxygen species (ROS) are in excess, and this might be due to excessive production or impaired elimination (26, 27). The anti-oxidative effect of KBs has been reported widely in both *in vivo* and *in vitro* studies, especially in the context of neuroprotection. ROS are mainly produced by mitochondria. Glutathione peroxidase (GSH-Px) is a key rate-limiting enzyme in the formation of ROS (28). During the normal process of oxidative phosphorylation, superoxide anion is generally produced at a lower concentration. When the mitochondria are damaged, the content of ROS increases when the calcium ions are overloaded, leading to excitotoxicity damage (29). Majority of the neuronal injuries are secondary to glutamate excitotoxicity, calcium overload, mitochondrial dysfunction, and oxidative stress.

KD lowers blood glucose levels and increases ketone production in the liver. The increase in KBs is mainly due to the oxidation of fatty acids, particularly the polyunsaturated fatty acids (PUFAs) (30). PUFAs activate peroxidase by blocking voltage-gated sodium and calcium channels, and regulate the membrane receptors in neurons or induce the expression of mitochondrial uncoupling protein (UCP) to increase. The uncoupling process reduces mitochondrial membrane potential, ultimately reducing the production of ROS (28, 31), (Figure 1).

It has been reported that the (D or L)-βHB scavenge ROS, while the ACA scavenges ROS species when their concentration exceeds physiological range (IC50 20–67 mM) (32). The beneficial effects on the redox potential of the electron transport chain is a common mechanism which is related to D-βHB (33). While all KBs (D/L-βHB and ACA) could reduce ROS accumulation and neuronal death which is triggered by the inhibition of glycolysis, but only D-βHB and ACA could prevent ATP declination in neurons (34–36). Conversely, in an *in vivo* model of hypoglycemia, where (D or L)-βHB prevented hippocampal lipid peroxidation, while ACA did not exhibit this effect (15, 32, 37–39). *In vivo* studies in mice that fed on KD suggested neuroanatomical variation with antioxidant capacity, and the most significant changes were observed in the hippocampus, as well as increased glutathione peroxidase level and total antioxidant capacities (40). In brain injury models, KD could activate the NF-E2-related factor 2 (Nrf2) pathway and then transported into the nucleus, followed by the expression of downstream antioxidant protein Heme oxygenase-1 (HO-1), which is considered to be one of the most essential substances for protecting against oxidative stress (41, 42). In a study with ischemic stroke model, ketone treatment after transient middle cerebral artery (MCA) occlusion enhanced the mitochondrial function, and reduced the oxidative stress, thus reducing the infarct volume, and improving neurological function after ischemic stroke. These neuroprotective effects were due to the upregulation of NAD+-dependent Sirtuin 3 (SIRT3) and its downstream substrates, superoxide dismutase 2 (SOD2) and forkhead box O3A (FOXO3A) in the penumbra area (43).

KD, ketone esters or βHB administration exerts neuroprotective effects as reported in models of a variety of neurological diseases (13–16, 44). In contrast, a recent study provided histopathological evidence of neurodegenerative progression that is related to KD in a transgenic mice model with impaired mitochondrial DNA repair, although there is an increase in mitochondrial biogenesis and antioxidant signatures (45). Other conflicting data suggested that exposure to high concentrations of KBs could elicit oxidative stress. A study in calf hepatocytes suggested that high doses of βHB or ACA could induce nitric oxide secretion, and lipid peroxidation, and reduced SOD, glutathione peroxidase and catalase expressions. Another study in rat hepatocytes showed that activation of mitogen-activated protein kinase (MAPK) pathway attributed to ACA, but not to βHB (46–48).

In summary, most of the previous reports associated KBs with the attenuation of oxidative stress, as they inhibited ROS production, prevented lipid peroxidation as well as protein oxidation, and increased the levels of antioxidant proteins. On the other hand, few other studies reported a correlation between KBs and induction of oxidative stress. Hence, it is necessary to consider that the antioxidative benefits conferred by KD might not be attributed to KBs themselves, and neuroprotective effects conferred by KBs might not be entirely attributed to anti-oxidative effects.

### Maintaining Energy Supply

Calorie intake is closely related to energy storage in the body. Protein and glucose produce 4,000 Kal/g, while fat produces 9,000 Kal/g, which has higher calorie value. This subsequently allows KD to stimulate mitochondrial biosynthesis, and increase UCP activity, which produces brain ATP and phosphoric acid, limiting the energy supply of patients (49). Increasing creatine concentration can improve the efficiency of cell metabolism, ultimately reducing the production of ROS while maintaining no significant changes in single cell metabolic output (50). This is because the mitochondrial damage and energy exhaustion are the critical factors in many neurological diseases (51–54). KD can increase metabolic efficiency and maintain the total metabolic amount stable under the conditions of relatively insufficient energy, thus enhancing the anti-injury ability of neurons.

Cerebral ketone metabolism significantly contributed to brain metabolism under conditions of energy challenges (55). Studies in suckling rats that rely on KBs as a necessary metabolic substrate in addition to glucose (56, 57) suggested a faster metabolic and behavioral recovery than adult rats with traumatic brain injury (TBI) (58). This led to the idea that such alternative substrates might have protective effects. The utilization of cerebral ketone metabolism as a therapeutic approach is not only feasible as it can bypass the early derangements of glucose metabolism after brain injury, but also improves metabolic efficiency (59, 60) and increases the ΔG' of ATP hydrolysis (61). A previous study demonstrated that intravenous infusion of ^14^C-3-βHB 3 h after brain injury in adult rats resulted in greater uptake of βHB as well as higher production of ^14^CO_2_ in the brain (62). Increased ketone metabolism subsequently improved the regional ATP concentrations, suggesting the potential for the alternative substrate as a therapeutic approach after cerebral injury (Figure 1).

ATP-sensitive potassium channel (K_ATP_) is a critical ion channel that links metabolism with electrical excitability and acts as a metabolic receptor (63, 64). K_ATP_ opens and closes depending on the intracellular ATP/ADP level. When the intracellular energy is insufficient, and ATP decreases, the channel opens up leading to potassium ion outflow, cell membrane hyperpolarization and decreased excitability; and when the intracellular energy is sufficient and ATP increases, the channel closes (65). Studies showed that ATP changes caused by glycolysis preferentially regulate the K_ATP_ activity (49, 66). In KD treatment, the glycolysis pathway was inhibited, reducing the energy produced by glycolysis (67). Thus, the ATP/ADP level was decreased, leading to the activation of K_ATP_, which in turn inhibit the seizure activity and reduce the excitatory injury (65).

**Figure 2:**
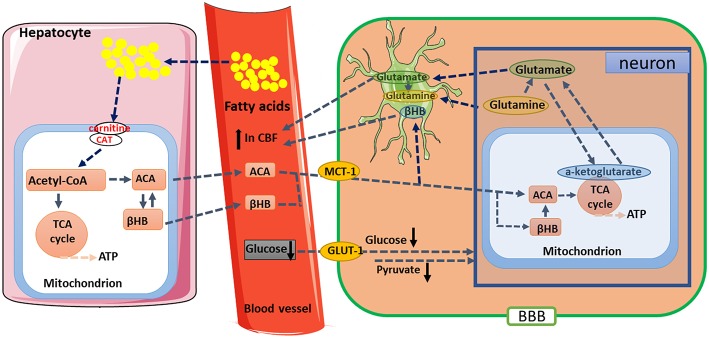
Proposed metabolic and hemodynamic changes induced by caloric restriction (CR). CR downregulated glucose metabolic pathway but upregulated ketogenic pathway. KBs are converted to acetoacetate (ACA) and then further to acetyl-CoA. The changes in metabolic pathway resulted in enhanced TCA cycle flux and glutamate-glutamine recycling between neurons and glial cells. Elevated CBF might be due to enhanced neuronal activities and increased levels of KBs. βHB, β-hydroxybutyrate; BBB, blood-brain barrier; CAT, carnitine acetyltransferase; GLUT-1, Glucose transporter 1; KBs, Ketone bodies; MCT1, monocarboxylate transporter 1; TCA, tricarboxylic acid.

In studies of the aging brain, multimetric neuroimaging was performed to characterize the caloric restriction (CR)-induced changes in brain metabolic and vascular functions in aging animals (68–70). The results showed that old rats (24 months of age) with CR reduced glucose uptake and lactate concentration, but increased the levels of KBs when compared with age-matched and young (5 months of age) controls. These metabolic changes were correlated with preservation of vascular function, where old rats with CR have maintained their cerebral blood flow compared to the age-matched controls. In the investigation of mitochondrial TCA cycle, citrate and α-ketoglutarate were found to be preserved in the old rats (71). These results suggested that CR has neuroprotective effects; and KBs, cerebral blood flow, and metabolism such as α-ketoglutarate might play an essential role in maintaining brain physiological functions during the process of aging (Figure 2). Thus, it is of profound implications to Understand the nutritional effects of KBs on brain function in the aging process and other age-related neurodegenerative disorders.

### Modulating the Activity of Deacetylation

Deacetylase plays an essential role in regulating cell survival, aging, apoptosis, and other physiological activities. Deacetylase involves histone deacetylase activity that is dependent on NAD. Histones are deacetylated to produce O-acetyl-ADP ribose and nicotinamide by using NAD+ as substrate. The latter, which acts as a signal factor, carries acetyl groups that are removed from the histones (72). Histones, which are modified significantly in post-translational stages, are important in regulating chromatin structure in eukaryotic cells. Lysine residues on histones are acetylated through acetyltransferases, which enables the unbound DNA to undergo transcription. Removal of such acetyl groups by histone deacetylases (HDACs) leads to tight binding of histones to DNA and results in transcriptional repression (73). HDAC inhibitors were previously reported as anti-inflammatory and anti-cancer agents (74), while recent data showed that they might also play certain roles in epileptogenesis (75). Valproic acid (VPA) is a broad-spectrum anti-seizure drug that is widely used clinically. It inhibits both classes I and class II HDACs which is cytotoxic to various types of cancers (76). Inhibition of HDAC might be an essential anti-epileptic mechanism of VPA (77).

Recent studies reported the inhibition of HDACs by βHB both *in vitro* and *in vivo*. These effects were found to be associated with an increase in resistance to oxidative stress (78–80). In particular, βHB induced acetylation of histone H3 lysine 9 (H3K9) and histone H3 lysine 14 (H3K14), as the transcription of genes was regulated by FOXO3A. In addition, *in vivo* administration of βHB via osmotic minipumps for over 24 h led to decreased carboxylation and decreased levels of 4-hydroxynonenal and lipid peroxides in the kidney. However, it should be noted that these effects in deacetylation modulation were not reported in brain cells or tissues. However, it might be reasonable to infer that the inhibition of HDACs by βHB and subsequent transcriptional changes might mediate some antioxidative effects in the brain, which are known to occur with KD (Figure 1).

However, evidence from a few other reports showed that KD and CR could activate deacetylation (72, 73). The expression of silence information regulator two related enzyme 1 (SIRT1) is usually up-regulated during neurodegeneration, playing a neuroprotective role (81, 82). There are various hypotheses regarding the neuroprotective mechanisms of SIRT1. This has a widespread deacetylase activity, with a variety of targets including histone, tumor suppressor P53, fork transcription factor (FOXO), DNA repair protein Ku70, peroxisome proliferator-activated receptor (PPARγ) and nuclear transcription factor-κB (NF-κB) (83). PPARγ can reduce the expression of inflammatory factors, especially the expression of NF-κB, thus alleviating the neuronal damage caused by excitotoxicity of N-methyl-D-aspartate (NMDA) (84).

### Modulating Inflammatory Responses

KBs could modulate inflammatory responses and functions of immune cells but have different and discrepant mechanisms (6). Prolonged nutrient deprivation could reduce inflammation (85). However, chronic ketosis that is induced in type 1 diabetes is considered a pro-inflammatory state (86, 87). The influence of βHB on inflammation is mainly because many cells in the immune system, including monocytes or macrophages, which express abundant GPR109A. While βHB exerts an effective anti-inflammatory response, high concentrations of KBs, especially ACA, might exert a pro-inflammatory effect (85, 88, 89). GPR109A, also known as hydroxy-carboxylic acid receptor 2 (HCA2), is a G protein-coupled receptor that is located on neutrophils, macrophages, adipocytes. In the brain, it is mainly found in the anterior cingulate cortex (90). Of GPR109A ligands plays anti-inflammatory roles in obesity, atherosclerosis, neurological diseases, inflammatory bowel diseases, and various types of cancer, which have been reported in several previous studies (91). GPR109A expression was found to be augmented in RPE cells of diabetic animals and patients (88, 89). Overexpression of GPR109A could enhance the anti-inflammatory effects of βHB in RPE cells, while genetic knockout or pharmacological inhibition of GPR109A could abrogate such effects (89). Rahman et al. (13) hypothesized that KD might exert its neuroprotective effects through βHB's actions on HCA2 receptors. The results showed that mice fed on KD or given βHB through subcutaneous minipumps had smaller ischemic infarct volumes following distal middle cerebral artery occlusion, and such effect was absent in HCAR2-null mice. βHB and exogenous nicotinic acid had anti-inflammatory effects in LPS or TNF-α-induced inflammation by decreasing pro-inflammatory proteins (i.e., COX-2, iNOS), or secreted cytokines (IL-1β, IL-6, TNFα, CCL2/MCP-1), partly by inhibiting NF-κB translocation (92). βHB decreases ER stress and NLRP3 inflammasome, activating anti-oxidative stress response (93). However, in neurodegenerative inflammatory responses, GPR109A-dependent protection mediated by βHB showed no involvement of the inflammatory mediators such as MAPK pathway signaling (e.g., ERK, JNK, P38), but may require COX-1-dependent PGD2 production (13, 88). It is interesting that in ischemic stroke animals, GPR109A in macrophage is required to play a neuroprotective role (13), but the inhibition of NLRP3 inflammasome by βHB in macrophages is found to be GPR109A independent (85). Although most of the previous studies associated βHB with anti-inflammatory effects, βHB might act as a pro-inflammatory factor and increase the markers of lipid peroxidation in calf hepatocytes (48). Whether βHB exerts anti-inflammatory or pro-inflammatory effects might depend on the cell types, βHB concentration, duration of exposure, and the presence of different co-modulators.

ACA might activate pro-inflammatory signaling, which is different from βHB. Increased level of ACA could intensify endothelial cell injury via NADPH oxidase/oxidative stress-dependent mechanism, especially when companies with high glucose concentration (87). High level of ACA in the umbilical cord of mothers with diabetes was associated with higher MCP-1 concentrations and protein oxidation rate (86). High ACA in diabetic patients was correlated with TNF-α expression. ACA was also found to be associated with the induction of MCP-1 expression, ROS accumulation and diminish cAMP level (94).

The underlying mechanisms still remain unclear whether ketones exert pro-inflammatory or anti-inflammatory effects. Additionally, due to the opposite effects of βHB vs. ACA on inflammation, the influence of ACA/βHB ratio on mitochondrial redox potential should be considered. The experiments to assess the effects of KBs on different cellular phenotypes and to compare the influences of ACA and βHB in different ratios, and at different concentrations should be performed in future studies.

## Applications of KD in Neurological Diseases Based on Neuroprotection

### Epilepsy

KD reduces oxidative damage and plays an anti-epileptic role. Mitochondria are the primary source of ROS/RNS. Complex I/III in the respiratory chain of ROS/RNS is sensitive to oxidative regulation (95). Previous studies revealed that epileptic seizures could lead to mitochondrial dysfunction and inhibit the activities of complexes I, II, and III (96, 97). KD can improve the inhibition of complex II/III, and significantly improve the oxidative stress in cytoplasm and mitochondria (98, 99). Haces et al. found that βHB and ACA could scavenge OH directly, and ACA could scavenge HOCL, ONOO-, and singlet oxygen directly *in vitro* (32). βHB and ACA were added to glutamic acid-containing neurons and calcium-containing mitochondria respectively, where both of these inhibited the accumulation of O_2_•- ions. In cortical sections of rats with acute seizures, these two ketones reduced cell death by inducing hydrogen peroxide. Greco et al. reported that super-physiological concentration of ketones could scavenge free radicals directly (100). In a further study, the concentration of βHB in plasma was 0.371 mM after 500 mg/kg of βHB was given to the hypoglycemic animal model, which significantly reduces the oxidative stress in the brain (32). In addition to scavenging free radicals directly, KD also induced the expression of antioxidant proteins. Both SOD1/2 and NQO1 can scavenge O_2_•- through Nrf2 signaling regulation (101–103). Nrf2 is activated by KBs and KD (104). Besides, the mitochondria in the hippocampus of rats fed with KD for more than 4 weeks were significantly higher than those in the control group. Therefore, KD might stimulate mitochondrial biosynthesis (49). After 10–12 days of KD feed, uncoupling proteins (UCPs) in the hippocampus of mice were increased, while the ROS produced by mitochondria was decreased (105). This suggested that KD can up-regulate mitochondrial UCPs. Fatty acids can also increase the expression of UCP, which may be due to the activation of transcription factors such as PPAR and FOX family. Recent studies showed that ACA and βHB could prevent neuronal death through oxidative stress mediation by mitochondrial permeability transition (mPT) activator. ACA and βHB have similar effects to that of cyclosporine A, an mPT blocker, which can increase the threshold of calcium-induced mPT opening (106). Continuous epileptiform activity decreased the Mg^2+^ in glial cells, depolarize cell membrane and opening of mPT, resulting in cell death (107). Cyclosporin A can inhibit this process and increase the survival rate of cells. Some scholars used KCNA1 mutant mice as epileptic models to observe the effects of KD and KBs on mPT pore and hippocampal long-term potentiation. The data showed that KBs have anti-epileptic and nootropic effects, and its anti-epileptic effect is directly related to mPT (108).

### Alzheimer's Disease

Alzheimer's disease (AD) is a multi-pathogenic neurodegenerative disease that is characterized by memory dysfunction, progressive cognitive impairment, visual-spatial skill impairment, executive dysfunction, personality, and behavioral changes. The main pathological changes include diffuse atrophy of cerebral cortex, neurofibrillary tangles, amyloid plaque deposition, loss of neurons and so on (109).

In recent years, more and more evidence showed that KD could effectively treat AD through various mechanisms. KD can enhance mitochondrial function and change glucose metabolism, reduce the production of advanced glycation end products (AGE) (110, 111). The accumulation of AGE during healthy aging accelerates the progression of AD. In several studies of AD model treated with low-dose USP methylene blue, which is described as pharmacological intervention, successfully increased mitochondrial respiration, memory enhancement and neuroprotective effects (112). KBs, especially βHB, can reduce the toxicity of 1-methyl-4-phenylpyridine (MPP+) to *in vitro* cultured neurons and the toxicity of amyloid protein fragment (Aβ) to hippocampal neurons (113), while KD can improve the electrophysiological function of the brain in AD mice (114).

Compared with the favorable data obtained from animal models, no definite conclusions have been drawn in clinical research. Henderson et al. in a randomized, double-blind, placebo-controlled, multicenter clinical trial demonstrated that KD can reduce oxidative stress and inflammation and delay the progression of AD, which is later manifested by improved cognitive function in AD patients (115). At present, clinical evidence for the therapeutic effects of KD on AD is still insufficient. It would be significant medical progress if the benefits of KD on this irreversible neurodegenerative disease could be elucidated.

### Parkinson's Disease

Parkinson's disease (PD) is the second common neurodegenerative disease following AD and the most common disorder associated with movement. It has been found that the progression of PD is related to inflammation (116, 117). There are several reactive human leukocyte antigens (HLA)-DR+ microglia in the substantia nigra of PD patients (118, 119). Toxins, pathogens, endogenous proteins or neuronal death can activate microglia, which might survive for a long time and self-renew due to positive feedback from the degenerated neurons. Activated microglia can release a variety of inflammatory factors, such as IL-1β, IL-6, TNF-α, IFN-γ, macrophage colony-stimulating factor, etc. and chemokines such as MIP-1α, MIP-1β, MCP-1 and prostaglandin E (120). Prostaglandin E can enhance the transmission between glutamatergic neurons through inhibition of astrocytes and re-uptake of glutamate, which is an excitatory neurotransmitter in the central nervous system, resulting in apparent excitatory neurotoxicity in the central nervous system (121, 122).

The mechanisms of anti-inflammation and inhibition of glutamate excitatory synapse transmission by KD can block this positive feedback, thus playing a therapeutic role. *In vitro* experiments have shown that KBs can improve mitochondrial respiratory chain dysfunction caused by exogenous complexes 1 and two inhibitors rotenone and 3-nitropropionic acid (123). According to a clinical study by Vanltallie et al. after 28 days of KD treatment, all PD patients showed moderate or more improvement in their symptoms. The score of MDS-UPDRS was reduced by 43% (124). As the sample size is too small, the clinical evidence of its conclusion further warranted confirmation in larger sample size. However, further research confirmed that the application of KD is expected to become a new strategy for the treatment of PD.

### Traumatic Brain Injury (TBI)

One of the early studies that explored the potential use of alternative metabolic substrates after TBI suggested that intravenous administration of βHB at 3 h following brain injury in adult rats led to higher uptake of βHB in the brain. The increased ketone metabolism subsequently increased the regional ATP concentrations, showing βHB as an alternative therapeutic substrate following TBI (62). In PND35 rats (analogous to an adolescent age group) fed on KD, plasma βHB levels increased within 6 h and sustained for a week. However, in adult rats, the plasma ketone levels showed no increase until 24 h after injury (125, 126). In PND35 rats, KBs significantly decreased the volume of the lesion and the number of degenerating fluoro-jade positive cells. The PND35 rats on the ketogenic diet also showed improvement in motor and cognitive function (127). However, this improvement showed no significance in adult rats, suggesting that the neuroprotective effects of KD on TBI were age-dependent. In order to play a neuroprotective role, it is necessary for KBs to enter the cerebral circulation in a short period. The age difference in the uptake of ketones might reflect the differences in the transporters or the timing of the increase in plasma substrates (128). Both factors might play a role in the effects of KBs in mitigating the cascades induced by TBI. Expression of monocarboxylate transporter (MCT) 1 and 2 are more abundant in the microvessels of PND35 rats than adult rats following TBI. This may, in turn, increase the uptake of ketones in the brain following injury (126).

Nevertheless, the plasma ketone concentrations in the adults are relatively delayed, which would delay the counteraction of ketones on pathological processes. In the adult brain, fasting for over 24 h was found to increase the plasma level of ketones levels and expression of MCTs (129). Besides, intravenous administration of βHB is considered as an alternative approach to KD. Considering the rapid pathological progression after TBI, it would be helpful to increase the availability and delivery of ketones (128).

## Summary and Perspectives

KD is used in the treatment of several neurological diseases for many years, and a large number of studies recently have validated the role of KD in neuroprotection. It could play the neuroprotective role by reducing oxidative stress, maintaining energy metabolism, modulating inflammation, modulating the activity of deacetylation, and other possible mechanisms. Although the specific mechanisms of KD in the treatment of neurological diseases are still uncertain, it is inevitable that all neurological diseases could affect human health through oxidative damage, energy metabolism disorders or inflammatory reactions. Neurological diseases often involve multiple mechanisms, and KD may also play a role through these mechanisms. In some diseases, such as epilepsy, AD, PD, KD can play a therapeutic role, while in some others, it plays a supporting role, facilitating the therapy of the disease, improving the symptoms and quality of life in patients. KD has excellent potential in clinical application, which further requires exploration. Future studies are necessary to further specify the roles of components in KBs and their therapeutic targets and related pathways, to optimize the strategy and efficacy of KD therapy.

## Data Availability

The raw data supporting the conclusions of this manuscript will be made available by the authors, without undue reservation, to any qualified researcher.

## Author Contributions

HY wrote the initial draft of the manuscript. WS provided both figures and made preliminary revision. FZ made preliminary revision. JW and QW made critical revision. All authors together planned the manuscript, critically revised the initial draft, and made final improvements before submission.

### Conflict of Interest Statement

The authors declare that the research was conducted in the absence of any commercial or financial relationships that could be construed as a potential conflict of interest.

## Abbreviations

ACA: acetoacetate
AD: Alzheimer's Disease
AGE: advanced glycation end products
BBB: blood-brain barrier
βHB: β-hydroxybutyrate
CAT: carnitine acetyltransferase
CR: caloric restriction
ETC: electron transport chain
FAO: fatty acid β-oxidation
FOXO3A: forkhead box O3A
GLUT-1: Glucose transporter 1
GSH-Px: glutathione peroxidase
HCA2: Hydroxy-carboxylic acid receptor 2
HDACs: histone deacetylases
HO-1: heme oxygenase-1
KBs: ketone bodies
KD: Ketogenic diet
MCT: monocarboxylate transporter
mPT: mitochondrial permeability transition
PD: Parkinson's Disease
PPAR: peroxisome proliferator-activated receptor
PUFAs: polyunsaturated fatty acids
RNS: reactive nitrogen species
ROS: reactive oxygen species
SOD: superoxide dismutase
TBI: traumatic brain injury
TCA: tricarboxylic acid
UCP: uncoupling protein.
